# Programmable kernel structures of atomically precise metal nanoclusters for tailoring catalytic properties

**DOI:** 10.1002/EXP.20220005

**Published:** 2023-04-10

**Authors:** Ya‐Hui Li, Shu‐Na Zhao, Shuang‐Quan Zang

**Affiliations:** ^1^ Henan Key Laboratory of Crystalline Molecular Functional Material, Henan International Joint Laboratory of Tumor Theranostical Cluster Materials, Green Catalysis Center and College of Chemistry Zhengzhou University Zhengzhou P. R. China

**Keywords:** atomically precise nanocluster, catalysis, kernel structure, structure‐reactivity relationships

## Abstract

The unclear structures and polydispersity of metal nanoparticles (NPs) seriously hamper the identification of the active sites and the construction of structure‐reactivity relationships. Fortunately, ligand‐protected metal nanoclusters (NCs) with atomically precise structures and monodispersity have become an ideal candidate for understanding the well‐defined correlations between structure and catalytic property at an atomic level. The programmable kernel structures of atomically precise metal NCs provide a fantastic chance to modulate their size, shape, atomic arrangement, and electron state by the precise modulating of the number, type, and location of metal atoms. Thus, the special focus of this review highlights the most recent process in tailoring the catalytic activity and selectivity over metal NCs by precisely controlling their kernel structures. This review is expected to shed light on the in‐depth understanding of metal NCs’ kernel structures and reactivity relationships.

## Graphical abstract

Compared to NPs, Ligand‐protected NCs with atomically precise structures and monodispersity have become an ideal candidate for understanding the well‐defined correlations between structure and catalytic property at an atomic level. Tuning the kernel structure of NCs to improve catalytic activity and selectivity by tailoring the number, types, and positions of metal atoms is a novel strategy.

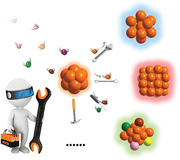

## 1 INTRODUCTION

In the modern world, catalysis plays the most important role in science and industry because approximately 90% of the chemical processes and two‐thirds of the chemical products involve at least one catalytic process.^[^ ^1^, ^2^, ^3^, ^4^, ^5^ ^]^ In recent years, catalysis science has advanced significantly mainly due to the rapidly growing nanotechnology and nanoscience.^[^ ^6^, ^7^, ^8^, ^9^ ^]^ Nanocatalysis, which catalyzes by nanoparticles (NPs), catches everyone's eyes because it unifies the advantages of both homogeneous catalysis and heterogeneous catalysis.^[^ ^10^, ^11^, ^12^ ^]^ Compared to their counterparts, metal NPs have unique electronic structures, quantum‐size effects, rich active surface atoms, and a large surface‐to‐volume ratio, showing widespread applications in heterogeneous catalysis.^[^ ^13^, ^14^, ^15^, ^16^, ^17^, ^18^, ^19^ ^]^ Unfortunately, these metal NPs are not uniform at the atomic level, and thus include multiple active sites, showing the property of multiple reaction pathways and low selectivity for most of the catalytic processes.^[^ ^20^, ^21^ ^]^ The polydispersity of NPs seriously affects the selectivity for desired products and hampers the identification of the active sites and reaction intermediates. Therefore, designing and synthesizing nanocatalysts with atomically precise structures is much needed.

Metal nanoclusters (NCs) protected by organic ligands, possess atomically precise structures and molecular purity.^[^ ^22^, ^23^, ^24^, ^25^, ^26^ ^]^ They have become a kind of star nanocatalysts for investigating the well‐defined relationships between structure and catalytic property.^[^ ^27^, ^28^, ^29^, ^30^, ^31^, ^32^, ^33^, ^34^, ^35^, ^36^, ^37^, ^38^, ^39^ ^]^ Metal NCs usually contain several to hundreds of metal atoms with core diameters ranging from 1 to 3 nm.^[^ ^22^ ^]^ These metal NCs show molecular‐like or non‐metallic behavior due to the quantized electronic structure arising from the quantum confinement effect, thus building a bridge between molecular catalysts and conventional metal NPs.^[^ ^40^ ^]^ With atomically precise structures in both the metal kernel and protected ligands, metal NCs are appropriate for the precise identification of active sites or reaction mechanisms by precisely modulating the core or surface structures while keeping other parts intact, which is difficult to achieve for metal NPs. So far, the regulation of the surface structure of metal NCs is usually related to ligand‐on, ligand‐off, and the type of ligands,^[^ ^41^, ^42^ ^]^ whereas kernel structures of metal NCs have been successfully tailored by the precise regulation of the number, type, and position of metal atoms, offering a new strategy for exploring the structure‐reactivity relationships at the atomic level.^[^ ^43^, ^44^, ^45^, ^46^, ^47^ ^]^ For example, three thiolated‐protected Au_25_(SC_6_H_13_)_18_, Au_38_(SC_6_H_13_)_24_, and Au_144_(SC_6_H_13_)_60_ exhibited size‐dependent CO_2_ reduction reaction (CO_2_RR) activity with high CO selectivity (> 90%) and long‐term stability, which improved with increasing the number of Au.^[^ ^48^ ^]^ Jin and co‐workers reported the shape effects of NCs on catalysis. Au_25_ nanospheres showed a higher CO_2_RR performance toward CO formation than that Au_25_ nanorods.^[^ ^49^ ^]^ More interestingly, Zhu and co‐workers removed or doped one atom with the precise location in the atomically precise metal NCs to modulate the catalytic performance.^[^ ^50^, ^51^ ^]^ Moreover, the mixed‐valence copper NCs [Cu_54_S_13_O_6_(*^t^* BuS)_20_(*^t^* BuSO_3_)_12_] (Cu_54_) supported on TiO_2_ exhibited excellent photocatalytic activity for phenol degradation under visible light.^[^ ^37^ ^]^ Therefore, these metal NCs provide an unprecedented opportunity to control catalytic performance and explore the catalytic mechanism of a catalyst by modulating the size, morphology, or even by an atom alteration.

The focus of this review is to discuss and highlight the structure‐performance relationship between the kernel structure of atomically precise NCs and catalytic activity and selectivity, and summarize the factors that affect the kernel structure of atomically precise NCs, such as size, structural isomerism, doping, addition and removal of single atoms, charge effects, etc. Therefore, we can tune the kernel structure of NCs by adjusting the number, type, and location of metal atoms, which further improves the catalytic activity and selectivity of NCs in catalytic reactions. This review can provide insightful guidance for designing NC catalysts with enhanced catalytic performance and provide theoretical models for studying the intermediate states of NC catalysts in catalytic processes.

## 2 KERNEL STRUCTURE MODULATES THE CATALYTIC PERFORMANCE OF METAL NCs

Compared with traditional nanocatalysts, atomically precise NCs can offer a better understanding of the catalytic mechanism at the atomic level.^[^ ^42^ ^]^ In general, the overall electronic structure of atomically precise metal NCs is susceptible to slight changes in the size, shape, dopant, and charge of their metal cores, which in turn leads to different catalytic reactivity and selectivity. Here, we discuss the important role of programmable kernel structures in atomically precise metal NCs for tailoring catalytic activity and selectivity by regulating size, shape, doping hetero‐atoms, vacancies, structural isomerism, charge effects, etc.

### 2.1 Size effect

**FIGURE 1:**
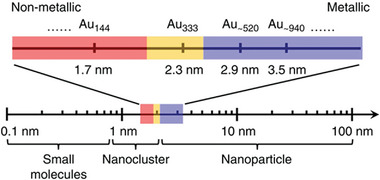
Schematic illustration of the evolution of size‐determined nanoparticles (red: nonmetallic or excitonic, yellow: transition state, blue: metallic or plasmonic). Adapted under the terms of the Creative Commons CC BY license.^[^ ^52^ ^]^ Copyright 2016, Springer Nature.

For a long time, it was a great challenge to explore the size‐dependent catalytic activity of NPs catalysts and the origin of this size dependence, due to the structural polydispersity and heterogeneity of the conventional NPs. The success in synthesizing a series of Au*_n_* (SR)*_m_* NCs with different sizes provides an excellent chance to explore the size‐dependent catalytic activity at an unprecedented atomic level.^[^ ^44^, ^45^, ^53^, ^54^ ^]^ As shown in Figure 1, Jin and co‐workers classified the Au NCs into three distinct states: metallic NCs with a core size larger than 2.3 nm (> Au_333_), transition NCs with a diameter of 2.3–1.7 nm (Au_333_ ∼ Au_144_), and nonmetallic NCs with a core size smaller than 1.7 nm (< Au_144_).^[^ ^40^, ^52^, ^55^ ^]^ They found that the Au NCs in the transition regime exhibited excellent catalytic activity in both CO oxidation and electrocatalytic alcohol oxidation compared with metallic and nonmetallic NCs.^[^ ^52^ ^]^ In addition, Au_144_ NC in the transition regime showed excellent catalytic performance and selectivity in both D‐glucose oxidation and methyl phenyl sulfide sulfoxidation, which was better than that of the metal NCs with a nonmetallic state.^[^ ^56^, ^57^ ^]^ Tsukuda and co‐workers investigated a series of Au*_n_* NCs (*n* = 10, 18, 25, 39) with atomically regulated sizes on hydroxyapatite for the aerobic oxidation of cyclohexane.^[^ ^58^ ^]^ The turnover frequency (TOF) of these catalysts increased with increased size, reaching the highest value at *n* = 39 (TOF_(Au_ *_39_*_)_ = 18 500 h^‒1^ Au atom^‒1^), and thereafter decreased with a further increased size to *n* = 85.

**FIGURE 2:**
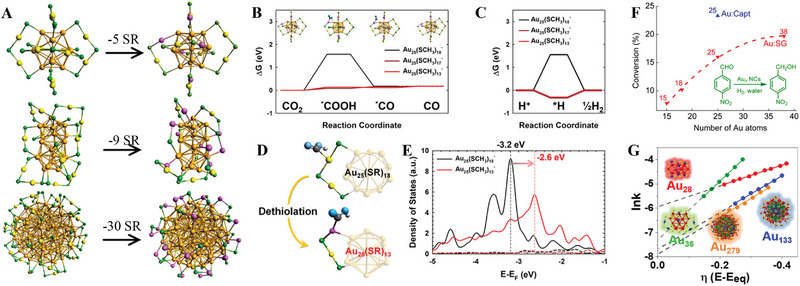
(A) Au_25_ (top), Au_38_ (middle), and Au_144_ (bottom). (B) Free energy diagrams (Δ*G*) of the CO_2_ reduction to CO. The inset indicated a possible active site. (C) Δ*G* of the H^+^ reduction to H_2_. (D) Illustration of the CO_2_ adsorption on the intact and dethiolated Au_25_ NCs. (E) Projected density of states of the sp‐states (dashed lines) and *d‐*states (solid lines) of the staple Au in the Au_25_(SCH_3_)_18_ (black) and Au_25_(SCH_3_)_13_ (red) NCs. Adapted with permission.^[^ ^48^ ^]^ Copyright 2021, Wiley‐VCH. (F) Schematic diagram of Au*_n_* (SG)*_m_* NCs as a catalyst for catalyzing the hydrogenation of 4‐nitrobenzaldehyde. Adapted with permission.^[^ ^59^ ^]^ Copyright 2014, American Chemical Society. (G) ln *k* vs overpotential plots of Au_28_ (red), Au_36_ (green), Au_133_ (blue), and Au_279_ (orange). Adapted under the terms of the Creative Commons CC BY license.^[^ ^60^ ^]^ Copyright 2018, American Chemical Society.

Recently, Lee and co‐workers reported that Au_25_(SC_6_H_13_)_18_, Au_38_(SC_6_H_13_)_24_, and Au_144_(SC_6_H_13_)_60_ NCs (short as Au_25_, Au_38_, Au_144_, respectively) (Figure 2A) showed size‐dependent CO_2_RR with high CO selectivity, following this order: Au_144_ > Au_38_ > Au_25_.^[^ ^48^ ^]^ Both the experiment and density functional theory (DFT) calculations indicated that the dethiolated Au site showed a relatively high charge density and upshifts of d‐states, which promoted the CO_2_RR by stabilizing the *COOH intermediate (Figure 2B–E). Thus, the authors concluded that the CO_2_RR performance of the Au NCs was confirmed by the number of dethiolated Au sites on the surface of these NCs. Furthermore, the surface of the metal NCs that is well related to the size of Au NCs provides an excellent opportunity for size‐dependent catalytic activity. For example, As shown in Figure 2F, Jin and co‐workers prepared various water‐soluble Au*_n_* (SG)*_m_* NCs (SGH = glutathione, Au_15_(SG)_13_, Au_18_(SG)_14_, Au_25_(SG)_18_, Au_38_(SG)_24_) and investigated their catalytic activity for the hydrogenation of 4‐nitrobenzaldehyde.^[^ ^59^ ^]^ All the Au*_n_* (SG)*_m_* catalysts exhibited ∼ 100% selectivity to 4‐nitrobenzyl alcohol, but their catalytic activity increased as their core size increased. DFT analysis revealed that the interaction between the ─CHO group of 4‐nitrobenzaldehyde and the Au(SR)_2_ staple motifs on the surface of Au*_n_* (SG)*_m_* leads to the difference in adsorption energies on different Au*_n_* (SG)*_m_*. Therefore, it can be concluded that the catalytic activity of these Au NCs is regulated by their surface area.

An opposite trend in that smaller Au NCs exhibited higher catalytic activity (Au_25_ > Au_38_ > Au_144_) was reported by Jin and co‐workers for styrene oxidation.^[^ ^61^ ^]^ They discovered that the size‐dependent catalytic activity is closely bound up with the quantum confinement effects of these Au NCs. Another example with the same trend was reported by Chen and co‐workers.^[^ ^62^ ^]^ They used various Au NCs with different sizes, including Au_11_Cl_3_(PPh_3_)_8_, Au_25_(PET)_18_ (PET = SCH_2_CH_2_Ph), Au_55_(PPh_3_)_12_Cl_6_, and Au_140_(S(CH_2_)_5_CH_3_)_53_, for electrocatalytic oxygen reduction reaction (ORR) in alkaline conditions and found size dependence in which Au_11_Cl_3_(PPh_3_)_8_ with smallest core size exhibits highest ORR catalytic performance. Recent theoretical studies demonstrated that the smaller the core diameter of Au NCs, the narrower the d bands become. And the d bands would shift toward the Fermi level. Thus, the authors claimed that the smaller the core size of Au NCs, the stronger the adsorption capacity of O_2_, which may be responsible for the variation of the ORR catalytic performance in these Au NCs. Notably, the ORR limiting current density of the Au NCs also followed this order and all the values are much higher than Au NPs with diameters greater than 2 nm. Furthermore, the same group observed the same ORR trend for Au_25_(PET)_18_, Au_38_(PET)_24_, and Au_144_(PET)_60_, with Au_25_(PET)_18_ having the best ORR performance.^[^ ^63^ ^]^ Chakraborty and co‐workers also investigated the size‐dependent ORR performance of 4‐tert‐butlylbenzenethiol (TBBT)‐protected Au NCs from 1 to 2.2 nm.^[^ ^60^ ^]^ Unlike the results reported by Chen's group, as shown in Figure 2G, the smallest NC Au_28_(TBBT)_20_ displays the highest overpotential of 540 mV to reach a specific current density and showed the worst selectivity due to the competition of the 2e^‒^ process. Although Au_36_(TBBT)_24_ has a similar core size to Au_28_(TBBT)_20_, it is the most effective ORR electrocatalyst as demonstrated by the minimum overpotential requirement (160 mV) and a complete 4e^‒^ pathway from O_2_ to H_2_O. Based on the overpotential, selectivity, and stability, the ORR performance decreases in the following sequence: Au_36_(TBBT)_24_ > Au_133_(TBBT)_52_ > Au_279_(TBBT)_84_ > Au_28_(TBBT)_20_. The more stable structure of Au_36_(TBBT)_24_ might be the main reason for the better ORR activity than Au_28_(TBBT)_20_ with a similar core size. Thus, the author indicated that even Au NCs with similar sizes can show striking distinction in catalytic performance just by changing a few Au atoms.

According to the above discussions, we can see that Au NCs provide an excellent platform to research the size‐dependent catalytic performance for various reactions. However, the size effect is usually not the only factor that determines the catalytic performance of NCs. Other contributing factors, such as the surface ligand, the number of active sites, and stability also can affect the catalytic performance.

### 2.2 Structural isomerism effect

**FIGURE 3:**
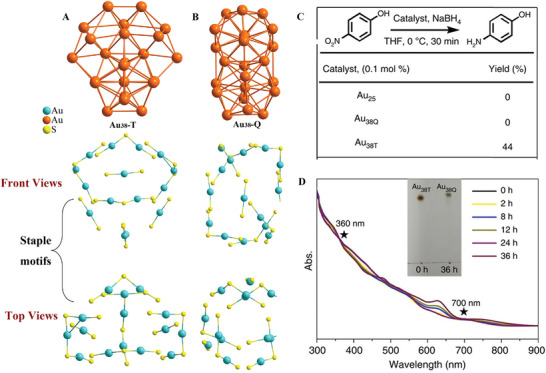
The Au_30_ kernel and the staple motifs in (A) Au_38_‐T and (B) Au_38_‐Q; (C) catalytic activities of Au_25_, Au_38_‐T, and Au_38_‐Q; (D) UV–vis‐NIR spectrum in toluene. Inset: Thin‐layer chromatography of Au_38_‐T before and after the transformation. Adapted under the terms of the Creative Commons CC BY license.^[^ ^70^ ^]^ Copyright 2015, Springer Nature.

Structural isomerism is a common phenomenon in organic chemistry on account of the diversity of carbon bonding. However, it is hugely difficult to investigate the structural isomerism effect at the nanoscale, due to the polydispersity of metal NPs. Nevertheless, many efforts have been made to search for the structural isomerism at the nanoscale, because this finding would offer excellent chances for deeply understanding the structure‐property relationship and guiding the design and synthesis of materials with unique functions. Metal NCs with precise compositions and structures are an ideal material to research structural isomerism at the atomic level.^[^ ^43^, ^64^, ^65^, ^66^, ^67^, ^68^ ^]^ The first pair of structural isomers Au_38_(PET)_24_‐Q and ‐T (Au_38_‐T/Q) were reported by Jin's group.^[^ ^69^, ^70^ ^]^ As shown in Figure 3A,B, Au_38_‐Q has a face‐fused bi‐icosahedral Au_23_ core, whereas the Au_23_ core of Au_38_‐T contains an icosahedra Au_13_ core and one Au_12_ cap via sharing two Au atoms. Furthermore, the surface structures of the two isomers are also quite different. Au_38_‐Q has three Au(SR)_2_ and six Au_2_(SR)_3_ staples to form the surface layer. The surface layer of Au_38_‐T contains three Au(SR)_2_, three Au_2_(SR)_3_, two Au_3_(SR)_4_ staple units as well as one bridging SR ligand. Interestingly, the two Au_38_ isomers exhibit different catalytic activities in reduction reactions. For instance, Au_38_‐T can reduce 4‐nitrophenol to 4‐aminophenol with a 44% yield, which is much higher than that of Au_38_‐Q (no reduction) under the same condition (Figure 3C). However, Au_38_‐T gradually converts to the thermodynamically stable Au_38_‐Q after 20 reuse cycles, and this transformation is irreversible (Figure 3D). Nevertheless, the results revealed the structural isomerism effect for catalytic properties at the nanoscale.

Zhang and co‐workers investigated the catalysis of two geometric isomers of Au_36_(DMBT)_24_ (DMBT = 3,5‐dimethylbenenethiol) NCs with different Au core arrangements but the same thiolate ligand.^[^ ^71^ ^]^ The intramolecular hydroamination of alkynes by Au_36_(DMBT)_24_ with a two‐dimensional (2D) arrangement of Au_4_ tetrahedral units (short for Au_36‐II_(DMBT)_24_) is better than that of Au_36_(DMBT)_24_ with a one‐dimensional (1D) arrangement of Au_4_ tetrahedral units (Au_36‐I_(DMBT)_24_). Experimental and computational studies demonstrated that the exposed Au sites of the two Au_36_(DMBT)_24_ catalysts favor distinct reaction intermediates and pathways. The exposed Au sites in the defective Au_36‐II_(DMBT)_24_ could indeed facilitate the hydroamination of 2‐ethylaniline by lowering the rate‐limiting activation barrier. While the defective Au_36‐I_(DMBT)_24_ binds to the exposed Au sites, which are vibrationally unstable and exhibit low catalytic activity. Two isostructural NCs (Ag_25_Cu_4_Cl_6_(dppb)_6_(PhC≡C)_12_(SO_3_CF_3_)_3_ and Ag_25_Cu_4_Cl_6_H_8_(dppb)_6_(PhC≡C)_12_(SO_3_CF_3_)_3_, abbreviated as Ag_25_Cu_4_ and Ag_25_Cu_4_H_8_, respectively, dppb = 1,4‐*bis*(diphenylphosphine)butane) were reported by Yuan et al.^[^ ^72^ ^]^ Compared with Ag_25_Cu_4_, the Ag_25_Cu_4_ kernel structure of Ag_25_Cu_4_H_8_ is distorted, and its Ag_13_ core is seriously expanded due to the participation of 8 hydrogen atoms. Moreover, Ag_25_Cu_4_ has 8 valence electrons and is generally considered to originate from the stable Ag_13_ core, so the metal atoms in the Cu_4_Ag_12_ shell should be in the +1 oxidation state. On the contrary, all metal atoms of Ag_25_Cu_4_H_8_ are +1 oxidation state. Ag_25_Cu_4_H_8_ reduced 4‐nitrophenol to 4‐aminophenol with up to 100% yield ascribed to the electron‐deficient Ag(I) and Cu(I) sites. While the conversion of Ag_25_Cu_4_ is only ∼ 8% under the same conditions due to the lower adsorption activity toward BH_4_^‒^.

**FIGURE 4:**
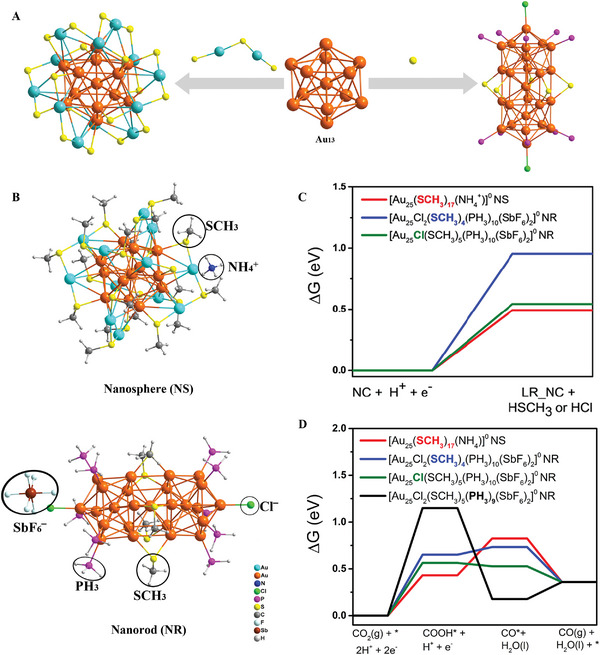
(A) Structures of Au_25_ sphere‐ and rod‐shape. (B) Structures of Au_25_ with the NH_4_^+^ and SbF_6_^‒^ as counterions. The proposed active sites of ligand removal are circled in black. Δ*G* values for removal of the ligand (C) and CO_2_ reduction to CO (D). Adapted with permission.^[^ ^49^ ^]^ Copyright 2018, American Chemical Society.

Besides, quasi‐isomers of metal NCs that have the same number of metal atoms but with different protected ligands are also discussed here, due to the rare structural isomers of metal NCs. Chen et al. reported a pair of Au_28_(S‐*c*‐C_6_H_11_)_20_ and Au_28_(TBBT)_20_ quasi‐isomers with the same Au_20_ kernel but completely different surface staple motifs. The different surface staple motifs of these two Au_28_ quasi‐isomers present different catalytic activities for CO oxidation.^[^ ^73^ ^]^ Liu et al. reported Cu_8_ clusters with one cube and two ditetrahedron‐shaped, exhibiting different catalytic performances of CO_2_RR.^[^ ^74^ ^]^ The results showed that the Cu_8_ cluster with ditetrahedron‐shaped exhibited higher activity and selectivity than the cube‐shaped Cu_8_ cluster at 1.0 V vs RHE (reversible hydrogen electrode) due to the lower free energy in the formation of the *COOH intermediate. This work provides a theoretical basis for atomically precise copper cluster models to elucidate the relationship between structure and catalytic performance, which lays the foundation for the study of copper‐based CO_2_RR electrocatalysts. Wang et al. reported a pair of Au_28_ (Au_28_(C_2_B_10_H_11_S)_12_(tht)_4_Cl_4_ and [Au_28_(C_4_B_10_H_11_)_12_(tht)_8_]^3+^ abbreviated as Au_28_‐S and Au_28_‐C, respectively, tht = tetrahydrothiophene) protected by carboranealkynyl and carboranethiolate.^[^ ^75^, ^76^ ^]^ The *COOH structure adsorbed by CO_2_* hydrogenation of Au_28_‐S has a low Δ*G* value, so it exhibits an excellent electrocatalytic reduction of CO_2_ reduction to CO (FE CO = 98.5%, FE = Faraday efficiency). Zhao et al. obtained two Au_25_ NCs of the same size but showed differences in atomic stacking (Figure 4A).^[^ ^49^, ^77^ ^]^ The Au_25_(SR)_18_ includes an icosahedral Au_13_ kernel and six Au_2_(SR)_3_ staple units, exhibiting a morphology of nanosphere. However, in Au_25_(PPh_3_)_10_(SR)_5_Cl_2_, two icosahedral Au_13_ kernels share one Au and are linked together via five bridging thiolates SR ligands at the waist part, forming a rod‐shaped morphology. Both two Au_25_ NCs are loaded on carbon black and applied as electrocatalysts for CO_2_RR. They found that the spherical Au_25_ NC exhibits higher current density and FE toward CO than those of the rod‐shaped Au_25_ NC at the whole range of applied potentials, indicating that the spherical Au_25_ NC processes better electrocatalytic activity for CO_2_RR than the rod‐shaped Au_25_ NC. The improved electrocatalytic performance can be attributed to the structural isomerism and negative charge state, which can stabilize the key *COOH intermediate of CO_2_RR (Figure 4B–D). Thus, this work suggested that the structural isomerism in Au NCs can affect the electrocatalytic activity, offering better insights into the electrocatalytic mechanism at the atomic level.

### 2.3 Doping effect

Doping has become one of the most essential strategies for modulating the intrinsic physical/chemical properties of metal NPs.^[^ ^78^ ^]^ In general, owing to the synergistic effect, doped metal NPs often exhibit improved catalytic properties than their monometallic counterparts.^[^ ^79^ ^]^ The improved catalytic performance of doped meter NPs is often influenced by the composition, the doping ratio as well as the distribution of the metallic components in the doped metal NPs.^[^ ^80^ ^]^ However, an in‐depth understanding of the precise synergistic interaction remains a huge challenge due to the polydispersity of metal NPs. Besides, the atomic‐level arrangement of the heterometals with specific numbers remains so far elusive. Metal NCs have broken new ground in precisely controlling composition and structure to tailor the intrinsic properties, owing to the uniform size and well‐defined structure.^[^ ^81^, ^82^ ^]^ Doping one or more foreign metals (such as Pd, Pt, Ag, Cu, Hg, and Cd) into Au NCs to tune their optical, electronic, and catalytic properties have been successfully achieved in many reports.^[^ ^83^, ^84^ ^]^ Interestingly, because of the differences in electronegativity and size, each foreign metal may prefer a different place in the parent NCs.^[^ ^85^, ^86^, ^87^ ^]^ For example, Pt and Pd atoms are often replaced with the center atom of the Au_25_ NCs, exhibiting enhanced catalytic activities for styrene and alcohol oxidation.^[^ ^88^, ^89^ ^]^ Although Cu and Ag atoms are located in the same group as Au, the Cu atom prefers to be doped at the icosahedral shell, whereas the Ag atom goes to the staple motifs.^[^ ^90^ ^]^ Here, we discuss the catalytic activity, selectivity, and stability of atomically precise alloy NC catalysts in the reaction system.

#### 2.3.1 Single heteroatoms doping effects

The introduction of single heteroatoms in alloy NCs offers a chance to investigate the mechanism of the reaction of catalytically active sites. Lee and co‐workers reported a single‐Pt–doped Au NCs (PtAu_24_(SC_6_H_13_)_18_, short as PtAu_24_) with excellent electrocatalytic activity and superior properties to commercial Pt/C catalysts in hydrogen evolution reaction (HER).^[^ ^91^ ^]^ Introducing a single Pt into the core can significantly alter the electronic structure of the parent Au_25_ NC and shift the reduction potential of PtAu_24_ NC positively by nearly 1 V in comparison with that of Au_25_ NC, which would lower the overpotential for reductive electrocatalysis. PtAu_24_ NC has a well‐matched reduction potential of HER and shows higher turnover frequencies (TOFs) of 4.8 mol_H2_(mol cat)^‒1^ s^‒1^ in tetrahydrofuran and 34 mol_H2_(mol cat)^‒1^ s^‒1^ in water at *η* = 0.6 V than any other HER catalysts under the same conditions. DFT results indicated that the energy change of the first step was thermodynamically neutral (−0.059 eV) for [PtAu_24_]^2–^, whereas it is + 0.539 eV for [Au_25_]^–^, indicating that the initial hydrogen binding is favored on [PtAu_24_]^2−^ but is endothermic on [Au_25_]^–^, which can explain the superior HER performance for PtAu_24_ NC. It also should be pointed out that the bond distance between the doped Pt and the adsorbed H is 1.788 Å, which is much smaller than that of the surface Au and the adsorbed H (2.031 Å), implying the stronger binding affinity between H and Pt. This work further established that doping a single Pt atom into Au NCs can modulate the redox potentials and binding affinity, offering a great chance to investigate the structure‐property relationships by only changing one atom. Chen and co‐workers reported another single‐Pt–doped PtAu_24_ NC with superior electrocatalytic performance for direct formic acid oxidation (FAO), showing a closely 34 times higher mass activity than that of the commercial Pt/C catalyst.^[^ ^92^ ^]^ The excellent FAO catalytic performance of PtAu_24_ NC can be also ascribed to the regulation of the electronic structure via doping a single Pt atom.

**FIGURE 5:**
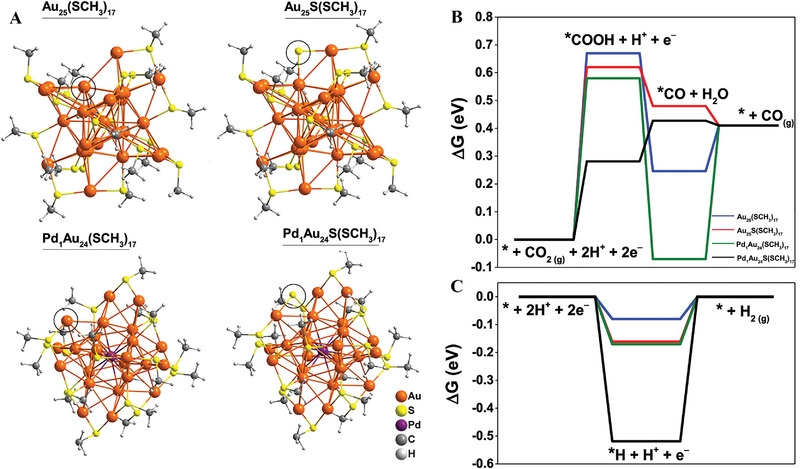
(A) The proposed active sites of NCs structures are circled in black. Δ*G* for electrochemical (B) CO_2_RR and (C) Hydrogen evolution reaction at *U* = 0 V vs RHE. Adapted with permission.^[^ ^93^ ^]^ Copyright 2020, American Chemical Society.

Very recently, Jin and co‐workers successfully prepared a single‐Pd–doped Au NC (PdAu_24_) as an electrocatalyst for CO_2_RR, showing a distinct doping effect compared to the parent Au_25_ NC (Figure 5A).^[^ ^93^ ^]^ The results showed that the PtAu_24_ NC was found to readily convert CO_2_ to CO with nearly 100% CO FE at large currents (−0.6 to −1.2 V versus RHE). However, the CO FE of the parent Au_25_ NC started to decrease at −0.9 V and dropped to 60% at −1.2 V due to the increasing evolution of H_2_ at large currents. To the best of our knowledge, full ligand‐protected NCs have no exposed active sites due to the tight coat of the ligands. Therefore, removing a small part −SR ligands or cleaving the organic −R moieties by thermal or chemical treatment to expose metal active sites or S sites of NCs is an effective means to improve catalytic activity.^[^ ^41^, ^94^ ^]^ As shown in Figure 5B,C, DFT results indicated that by removing the ligands explored the S sites of the thiolate ligand in the surface layer are the active sites for CO_2_RR, whereas the Au sites are the active sites for H_2_ evolution. Doping a single Pd atom in the core of Au_25_ NC is beneficial for the retention of the S site in the ligand removal process under cathodic potentials, thus inhibiting H_2_ evolution at large currents. This work again demonstrated that the catalytic performances of Au NCs can be tailored by replacing only one foreign atom. In addition, a lot of works have investigated the doping effect of Au_25_ NC, indicating that Au_25_ NC gives a new insight into researching the structure‐property relationship by doping foreign atoms.

**FIGURE 6:**
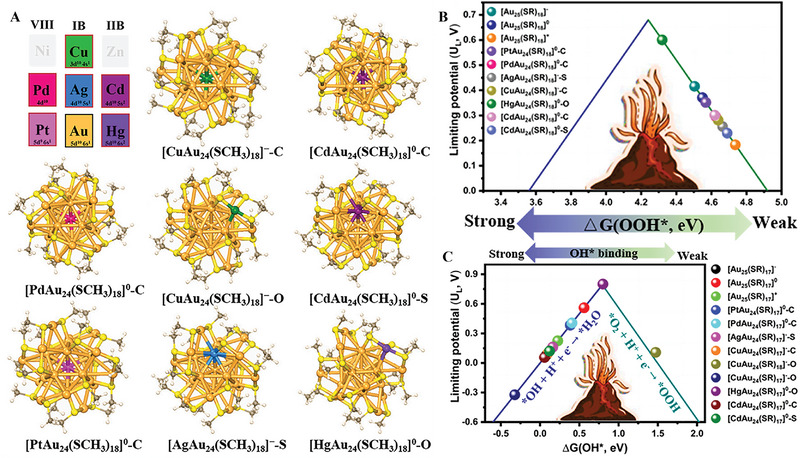
(A) Optimized structures of [MAu_24_(SCH_3_)_18_]*^q^* clusters. (B) Volcano relation between *U*_L_ and ΔG_*OOH_ for NCs. (C) Volcano plot correlation of the limiting potential as a function of ΔG_*OH_. Adapted with permission.^[^ ^95^ ^]^ Copyright 2021, American Chemical Society.

In 2021, Tang and co‐workers presented a comprehensive investigation of single‐M–doped [MAu_24_(SR)_18_]*^q^* NCs (Figure 6A, M = Cu, Pd, Ag, Cd, Pt, and Hg) with M‐exposure and S‐exposure systems as electrocatalysts for ORR through theoretical calculations.^[^ ^95^ ^]^ Theoretical studies indicated that the most favorable pathway for the fully ligand‐protected clusters was the formation of H_2_O_2_, whereas the partially ligand‐removed clusters prefer H_2_O as the final product through the 4e^−^ pathway. The fully protected [HgAu_24_(SCH_3_)_18_]^0^‐O and partially ─SCH_3_ removed [HgAu_24_(SCH_3_)_17_]^0^‐O exhibited the best ORR activity to produce H_2_O_2_ and H_2_O among the single‐M–doped MAu_24_ NCs. From the Volcano relation as shown in Figure 6B,C, the authors explained the key factors of selectivity and activity for ORR by correlating the adsorption energy of the intermediates in the ORR process and the active metal d‐band center. In comparison to the strong d‐electron effect in Au_25_ *^q^* and other doped MAu_24_ *^q^*, the staple–doped Hg atom shows a strong s‐electron effect, rendering it an excellent ORR electrocatalyst. These results demonstrated that modulating the electronic structures of metal NCs tailors the catalytic activity by doping a metal atom. Except for Au_25_ NCs, single‐atom doping can be also achieved in other metal NCs.^[^ ^96^ ^]^ For example, an Au_8_Pd NC exhibited excellent catalytic performance for benzyl alcohol oxidation reported by Zhu and co‐workers, whereas Au_9_, Au_24_Pd, and Au_25_ NCs cannot.^[^ ^97^ ^]^ The authors concluded that the Pd site in Au_8_Pd NC served as the unique active site in the entire reaction system. The elegant work from the same group investigated single‐M–doped MAg_24_(SPhMe_2_)^−^ NCs and further studied the center‐doping effect of catalytic capability in the carboxylation reaction of CO_2_.^[^ ^51^ ^]^ The foreign atoms indeed enhanced the catalytic performance, which follows as Au@Ag_24_ > Pd@Ag_24_ ≈ Pt@Ag_24_ > Ag@Ag_24_. The electron density on the Ag atoms decreased owing to the electron donation from the external Ag atoms to the foreign atoms, and the degree of the electron donation depended on the properties of the doped atoms. The enhanced catalytic performance was attributed to the more positive charge states of the MAu_24_ NCs with a higher chemical adsorption capacity of CO_2_. Furthermore, the central doping of foreign atoms into Ag_25_ NC can bring a significant enhancement in stability.

#### 2.3.2 Alloy effects

Li and co‐workers studied the doping effects of metal NC catalysts using Pt, Ag, and Cu–doped M*_x_* Au_25‐_ *_x_* (SR)_18_ NCs and Au_25_ (Pt, *x* = 1; Ag, *x* = 0–5; Cu, *x* = 0–6). These NCs displayed distinct catalytic performance on the carbon‐carbon coupling reaction of *p*‐iodoanisole and phenylacetylene.^[^ ^98^ ^]^ Compared to the parent Au_25_(SR)_18_, the single‐Pt–doped PtAu_24_(SR)_18_ NC showed a decreased catalytic activity but a retained selectivity, whereas the overall catalytic performance of Ag*_x_* Au_25‐_ *_x_* (SR)_18_ NCs is comparable to Au_25_(SR)_18_. More interestingly, Cu*_x_* Au_25‐_ *_x_* (SR)_18_ NCs prefer to produce 4,4′‐dimethoxy‐1,1′‐biphenyl through the Ullmann homo‐coupling pathway, which is different from the other three metal NCs. The conversion of *p*‐iodoanisole was mainly ascribed to the electronic effect in the 13‐atom core of the doped bimetallic NCs, while the selectivity is largely affected by the variety of metal atoms on the M*_x_* Au_12‐_ *_x_* shell in the bimetallic NCs. In another work also reported by Li and co‐workers, Cu*_x_* Au_25‐_ *_x_* (SR)_18_ NCs (*x* = 0 − 5) showed an enhanced selectivity for the catalytic oxidation of styrene but without clearly altering the catalytic activity, while Ag*_x_* Au_25‐_ *_x_* (SR)_18_ NCs (*x* = 4 − 8) displayed both enhanced catalytic activity and selectivity to benzaldehyde.^[^ ^99^ ^]^ These works provided unique ideas into the doping effects of metal NCs on the catalytic performance and selectivity in various oxidation reactions.

Jin and co‐workers successfully synthesized the heavily Ag–doped metal NC [Ag*_x_* Au_25−_ *_x_* (SC_6_H_11_)_18_]^−^ (*X* = 24) loaded on a carbon black support and exhibited superior electrocatalytic performance for ORR in an alkaline environment compared to lightly doped Au clusters.^[^ ^100^ ^]^ The authors pointed out that one of the key factors influencing catalytic efficiency is the catalytic site. Yao and co‐workers quickly synthesized atomically monodisperse Au_25_Ag_2_ using the antigalvanic reduction method.^[^ ^101^ ^]^ The main structure of Au_25_ in Au_25_Ag_2_ is still maintained, while Ag_2_ is only incorporated into the surface of Au_25_. Compared with Au_25_, Au_25‐_ *_x_* Ag*_x_* can be kept for a week without change after exposure to air and light and also exhibited accelerated hydrolysis of 1,3‐diphenylpropyl‐2‐ynyl acetate with a conversion rate of 52% under the same conditions, which is mainly attributed to the two silver atoms on Au_25_ acting as active sites. This work provided insights into a better understanding of heteroatoms as active sites in nanoclusters.

**FIGURE 7:**
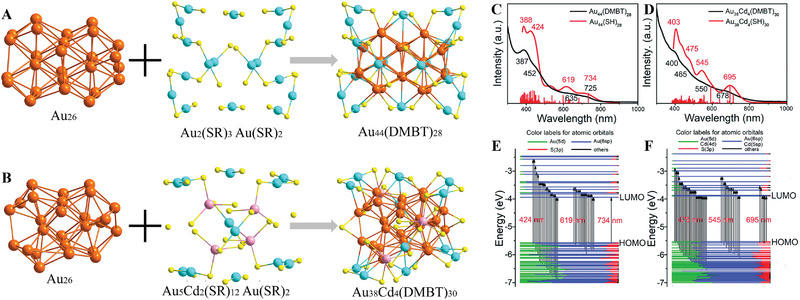
Structural analysis of (A) Au_44_(DMBT)_28_ and (B) Au_38_Cd_4_(DMBT)_30_ with the Au_26_ kernels and various motifs. UV–vis‐NIR spectra of (C) Au_44_(DMBT)_28_ and (D) Au_38_Cd_4_(DMBT)_30_. Molecular orbital energy level diagrams for (E) Au_44_(DMBT)_28_ and (F) Au_38_Cd_4_(DMBT)_30_. Adapted under a Creative Commons Attribution 3.0 unported license.^103^ Copyright 2021, The Royal Society of Chemistry.

Because Cd has one more valence electron than Au, the introduction of Cd usually results in the surface reconstruction of gold NCs and further modulates the physicochemical properties of NCs.^[^ ^33^ ^]^ Interestingly, a rare Cd–doped Au NC [Au_13_Cd_2_(PPh_3_)_6_(PET)_6_(NO_3_)_2_]_2_Cd(NO_3_)_4_ (abbreviated as Au_26_Cd_5_) exhibited superior catalytic performance and excellent recyclability for A^3^‐coupling reaction, whereas the Au_25_(PET)_18_ NC showed no catalytic activity for A^3^‐coupling reaction under the same conditions.^102^ The authors concluded that the excellent catalytic performance of Au_26_Cd_5_ is greatly affected by the synergy between the foreign Cd atoms and the adjacent Au atoms on the surface of the Au_13_ core. Meanwhile, NCs with rich electronic configurations due to the number of dopants have attracted extensive attention. Moreover, Liu and co‐workers reported an Au_38_Cd_4_(DMBT)_30_ bimetallic NC, which was synthesized by Cd‐induced surface recombination of Au_44_(DMBT)_28_ (Figure 7A,B).^103^ Au_38_Cd_4_(DMBT)_30_ contains an FCC Au_26_ kernel which is slightly distorted from “slender” to “stout” compared to the kernel of the parent Au_44_(DMBT)_28_. While the two Au_5_Cd_2_(DMBT)_12_ staple motifs of Au_38_Cd_4_(DMBT)_30_ are formed by replacing four Au_2_(DMBT)_3_ stable motifs in Au_44_(DMBT)_28_ with two Au_5_Cd_2_(DMBT)_12_. As shown in Figure 7C–F, compared to Au_44_(DMBT)_28_, surface modification of Cd atoms in Au_38_Cd_4_(DMBT)_30_ causes an obvious change in the electronic structure. In particular, the Au_38_Cd_4_(DMBT)_30_ bimetallic NC exhibits better visible‐light‐driven catalytic decomposition of methyl orange compared to the parent Au_44_(DMBT)_28_ catalyst. This work offered a solid theoretical foundation for studying the electronic structure of NC to alter catalytic performance at the atomic level.

Cheng and co‐workers explored the catalytic performance of three metal–doped Ag clusters (Ag_4_M_2_(SR)_8_, M = Ni, Pd, Pt; SR = SPhMe_2_; abbreviation as Ag_4_M_2_) with a distorted hexahedron structure composed of eight sulfur atoms as vertices.^[^ ^104^ ^]^ Four silver atoms are at the waist, and two heteroatoms are at the center of the top and bottom. The highest occupied molecular orbital and lowest unoccupied molecular orbital (LUMO) gaps of Ag_4_Ni_2_, Ag_4_Pd_2,_ and Ag_4_Pt_2_ are 1.60, 2.24, and 2.61 eV, respectively, due to doping metal atoms. Therefore, the three Ag_4_M_2_ NCs have opposite photocatalytic degradation effects on methyl orange and rhodamine B, which was attributed to the difference in electronic structure.

The unique surface structures in alloy NCs provide a chance to study the structure‐activity relationship between structure and catalytic performance. Wang and co‐workers successfully synthesized the largest Ag‐Au alloy NC [Ag_46_Au_24_(SR)_32_](BPh_4_)_2_ (R = ^t^Bu, Ag_46_Au_24_), which was assembled by three layers of bimetallic Ag_2_@Au_18_@Ag_20_ kernel stabilized by doped Ag_24_Au_6_(SR)_32_ shell.^[^ ^105^ ^]^ In styrene oxidation, the styrene conversion of homogold Au_25_/CNT is 72.8%, which is higher than that of homosilver Ag_44_/CNT (43.6%). However, Ag_44_/CNT shows a much higher selectivity (92.6%) than Au_25_/CNT (66.4%) for benzaldehyde. Interestingly, surface–doped bimetallic Au_24_Ag_46_/CNT exhibits both better conversion ∼ 70% and higher selectivity > 95%, which can be attributed to the synergistic effect of gold and silver.

**FIGURE 8:**
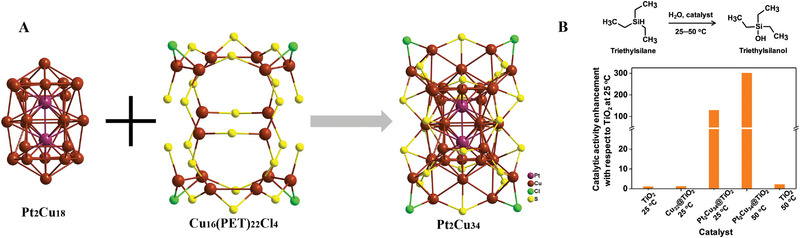
(A) Structures analysis of Pt_2_Cu_18_, Cu_16_(PET)_22_Cl_4_, and Pt_2_Cu_34_ NCs. (B) Catalytic oxidation of silane to silanol (top). Compared to the TiO_2_ support at 25°C, catalytic activity enhancement of Cu_32_@TiO_2_ and Pt_2_Cu_34_@TiO_2_. Adapted with permission.^[^ ^106^ ^]^ Copyright 2021, American Chemical Society.

Although some alloy NCs have been reported, the synthesis of heteroatom–doped NCs was much more difficult than that of homometallic NCs, which was ascribed to the atomic size effect of heterometals (different period and electron number in the outer layers). Lee and co‐workers synthesized Pt–doped copper NC [Pt_2_Cu_34_(PET)_22_Cl_4_]^2−^ (Pt_2_Cu_34_) by a metal exchange strategy using the [Cu_32_(PET)_24_Cl_2_H_8_]^2−^ (Cu_32_) precursor.^[^ ^106^, ^107^ ^]^ Pt_2_Cu_34_ contains an abnormally interpenetrating incomplete double icosahedral core (Pt_2_Cu_18_) bridged by a Cu_16_(PET)_22_Cl_4_ shell (Figure 8A). Cu_32_ has a bisquare antiprismatic Cu_14_H_8_ core protected by two Cu_7_(PET)_11_Cl and two Cu_2_PET motifs. Compared with Cu_32_, Pt_2_Cu_34_ has the loss of hydrides and retained 2‐electrons due to the introduction of large‐sized Pt atoms, while the total number of metal nuclei and free valence electrons also changed, increasing from 32 to 36 and from 0 to 10, respectively. The Pt atoms located in the core of NCs exhibit a doping‐induced synergistic effect that will facilitate the catalytic oxidation of silane to silanol (Figure 8B).

### 2.4 Single‐atom dislodging/adding effects

Imaoka and co‐workers succeeded in realizing platinum clusters Pt_12_ and Pt_13_ with well‐defined numbers of metal atoms by using dendrimer ligands as macromolecular templates.^[^ ^108^ ^]^ Misshapen Pt_12_ NC is formed when only one atom was removed from the magic number NC Pt_13_, while its ORR catalytic performance is remarkably enhanced. The excellent ORR catalytic performance of Pt_12_ may be attributed to the fact that the binding energy of Pt_12_ is ideal between 0.2–0.3 eV, while the binding energy of Pt_13_ with icosahedron is too strong. Therefore, metastable NCs with deformed coordination can generate high activity compared to NCs with well‐known icosahedral atomic coordination.

**FIGURE 9:**
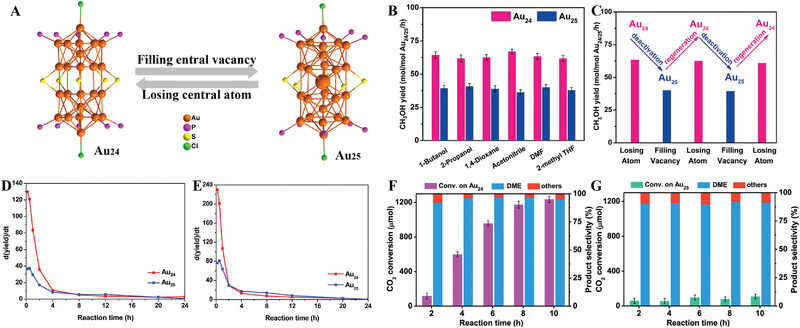
(A) Atomic structures, (B) catalytic performance, and (C) activity reversible behavior of Au_24_ and Au_25_. Adapted with permission.^[^ ^109^ ^]^ Copyright 2019, Wiley‐VCH. Intramolecular cyclization of (D) 5‐hexyn‐1‐amine and (E) 2‐ethnylaniline over the Au_24_ and Au_25_. Adapted with permission.^[^ ^50^ ^]^ Copyright 2019, The Royal Society of Chemistry. The catalytic activity of CO_2_ hydrogenation over (F) Au_24_/SiO_2_ and (G) Au_25_/SiO_2_ catalysts. Adapted with permission.^[^ ^110^ ^]^ Copyright 2020, American Chemical Society.

Removal or addition of central atoms can significantly tune the electronic structure of the NC catalysts and enhance or lose the catalytic activity for methane oxidation. Cai and co‐workers prepared two Au NCs [Au_24_(PPh_3_)_10_(PET)_5_Cl_2_]^+^ (Au_24_) and [Au_25_(PPh_3_)_10_(PET)_5_Cl_2_]_2_^+^ (Au_25_) to investigate the effect of only one Au atom on methane oxidation (Figure 9A–C).^[^ ^109^ ^]^ Au_24_ processing an internal vacancy due to the loss of an Au atom in the center of Au_25_ exhibits more efficient performance for methane oxidation but poorer stability compared with Au_25_, which is mainly attributed to the structural rearrangement of Au_24_ transforms into Au_25_. However, the destroyed Au_24_ (actually Au_25_) can be converted into Au_24_ by reacting with excess triphenylphosphine. Therefore, Au_24_ and Au_25_ can be used as theoretical models to switch on/off the activity of NC catalysts by shuttling in/out only one atom.

Furthermore, the same Au_24_ NC also exhibits excellent intramolecular hydrogenation of alkynes (Figure 9D,E)^[^ ^50^ ^]^ as well as CO_2_ hydrogenation to diethyl ether (Figure 9F,G).^[^ ^110^ ^]^ The potential barrier of TS‐Au_24_ (TS: transition state for catalyst activation upon adsorption) is 1.2 kcal mol^‒1^ smaller than that of TS‐Au_25_, which is considered to be the key reason for the high activation of Au_24_ in the intramolecular hydrogenation of alkynes due to shuttling effect of one gold atom. In the hydrogenation reaction of CO_2_, Au_24_ catalyst with internal vacancy has the characteristics of protecting the structural relaxation and inhibiting aggregation, and retaining the catalytic activity. Thus, Au_24_ with breath‐like motions exhibits good catalytic recyclability. Therefore, the totally different stability and catalytic activity of Au_25_ and Au_24_ in different catalytic systems indicate that catalytic mechanisms and catalytic conditions are quite important for NC catalysts.

Sun et al. reported two amidinate‐protected stable copper hydride clusters (Cu_11_H_3_(Tf‐dpf)_6_(OAc)_2_ (Cu_11_) with three interstitial μ_6_‐H and Cu_12_H_3_(Tfdpf)_6_(OAc)_2_]·OAc (Cu_12_) with three interstitial μ_5_‐H, Tf‐dpf = *N*,*N*′‐Di(5‐trifluoromethyl‐2‐pyridyl)formamidinate).^[^ ^32^ ^]^ The core of Cu_11_ is composed of edge‐sharing rectangular pyramids that share two copper atoms. However, the core of Cu_12_ contains three face‐sharing octahedra, in which 12 copper atoms have a hexagonal close‐packed structure with ABAB stacking mode. Cu_11_ exhibits higher catalytic activity than Cu_12_ in the reduction of 4‐nitrophenol to 4‐aminophenol. The structure of copper hydride clusters and the coordination modes of hydrides are different due to one copper atom, which further proves that the coordination mode of hydrides is the key to efficient catalysis. Shen et al. successfully synthesized two bidentate NHC ligands and PA (phenylacetylide) co‐protected Au_16_ and Au_17_ with the same Au_13_ core.^[^ ^111^ ^]^ Au_16_ was formed by the Au_13_ core surrounded by three NHC‐Au‐PA OMs (organometallic motifs = OMs), one of which is independently uncoordinated. While the structure of Au_17_ was composed of four OMs coordinated to the Au_13_ core, of which there are two independent OMs. Au_17_ was 1.7 times more effective than Au_16_ in the hydroamination of alkynes under the same conditions, which was mainly because Au_17_ has one more independent OM than Au_16_.

### 2.5 Effect of the same motifs but different core

**FIGURE 10:**
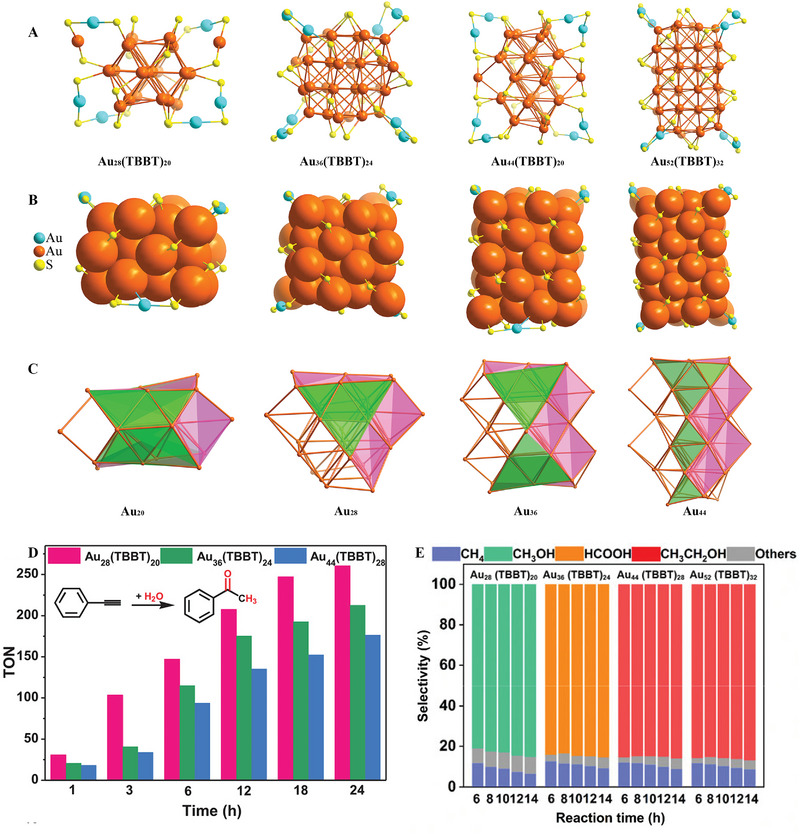
(A,B) Atomic structures of Au_8_ *_n_*_+4_(SR)_4_ *_n_*_+8_ NCs; (C) Au with (111) (green) and (100) (pink) faces. (D) Catalytic performances of the Au_8_ *_n_*_+4_(SR)_4_ *_n_*_+8_ catalysts for the hydration of phenylacetylene, Adapted with permission.^[^ ^112^ ^]^ Copyright 2019, Wiley‐VCH; and (E) CO_2_ hydrogenation. Adapted with permission.^[^ ^113^ ^]^ Copyright 2020, Springer Nature.

Proving catalytic active centers is a key step in understanding the reaction mechanism in the catalytic process, and exploring the active sites of NC catalysts has always been a research hotspot. Interestingly, when the surface protection nodes of the NCs are the same, whether the differences in their internal structures can affect the catalytic performance is a topic worthy of research by scientists. Sun and co‐workers reported a series of Au_8_ *_n_*_+4_(SR)_4_ *_n_*_+8_ with FCC structure (SR = 4‐tert‐butylthiophenol, *n* = 3, 4, 5, Au_28_(SR)_20_, Au_36_(SR)_24_, Au_44_(SR)_28_). Au_8_ *_n_*_+4_(SR)_4_ *_n_*_+8_ NCs have the same surface motifs and periodically arranged cores with four (111) faces protected by dimer motifs and (100) faces (8 for Au_28_, 12 for Au_36_, and 16 for Au_44_) covered by bridging thiolates (Figure 10A–D).^[^ ^112^ ^]^ These catalysts exhibited 100% selectivity for the formation of acetophenone during the hydration of phenylacetylene. However, the catalytic activity was as follows Au_28_(SR)_20_ > Au_36_(SR)_24_ > Au_44_(SR)_28_. In addition, the apparent activation energy of Au_8_ *_n_*_+4_(SR)_4_ *_n_*_+8_ is in the following sequence: Au_28_(SR)_20_ < Au_36_(SR)_24_ < Au_44_(SR)_28_, which further proves that Au_28_(SR)_20_ has excellent catalytic activity for alkyne hydration. Therefore, the catalysts have different core structures and electron‐accepting abilities resulting in the difference in affinities for alkynes. At the same time, (Au_8_ *_n_*_+4_(SR)_4_ *_n_*_+8_ (*n* = 3, 4, 5, 6, Au_28_(SR)_20_, Au_36_(SR)_24_, Au_44_(SR)_28_, Au_52_(SR)_32_) as a heterogeneous catalysts also exhibit excellent catalytic activity CO_2_ hydrogenation with different selectivity (Figure 10E).^[^ ^113^ ^]^ Interestingly, the Au_36_(SR)_24_ produces formic acid as the main product, whereas Au_28_(SR)_20_ exhibits better methanol selectivity. The final product of Au_44_(SR)_28_ or Au_52_(SR)_32_ with a larger core is ethanol. This study provides a new idea for designing atomically precise catalysts by controlling the different core structures but the same motifs of gold nanoclusters to tune the performance of CO_2_ conversion.

### 2.6 Effect of charge state

It is well known that the charge state of NCs will impact their electronic structure and change local bond length, which in turn determines their catalytic performance.^[^ ^114^ ^]^ Au_25_ with Au_13_ core protected by different ligands as star NCs has been widely studied in catalytic reactions of electrocatalytic CO_2_ reduction,^[^ ^115^, ^116^, ^117^ ^]^ aerobic oxidation,^[^ ^118^ ^]^ hydrogenation,^[^ ^119^, ^120^ ^]^ coupling reactions,^[^ ^121^ ^]^ and electrocatalytic water splitting,^[^ ^122^ ^]^ etc. However, the mechanism of Au_25_ with different charge states in the catalytic reaction process remains to be further explored. Kauffman and co‐workers reported atomically precise Au_25_(PET)_18_ (abbreviated Au_25_ *^q^*, *q* = −1, 0, +1) NCs with a different state of charges to understand how charged active sites work in electrochemical reactions of CO_2_ reduction to CO.^[^ ^123^ ^]^ Negatively charged Au_25_^−^ exhibits enhanced CO_2_ reduction activity due to its ability to stabilize CO_2_ + H^+^ co‐adsorption. The positively charged Au_25_^+^ plays an important role in stabilizing CO + OH^−^ co‐adsorption and exhibits enhanced CO oxidation activity. Finally, the stronger binding of the positively charged Au_25_^+^ to the OH^−^ reaction product leads to reduced O_2_ reduction activity. The results indicated that the role of NCs with charged active sites in electrocatalytic reactions can be tuned by electronic structure. Chen and co‐workers also researched the effect of the charge state of [Au_25_(SC_12_H_25_)_18_]*^q^* (*q* = −1, 0 and +1) NCs on the electrocatalytic activity of H_2_O_2_ from O_2_.^[^ ^124^ ^]^ The yield of [Au_25_(SC_12_H_25_)_18_]*^q^* as a catalyst for electrocatalytic oxygen to hydrogen peroxide can reach ∼ 90%, which is caused by the electron transfer from the anion Au_25_ core to the LUMO (π*) of O_2_.

**FIGURE 11:**
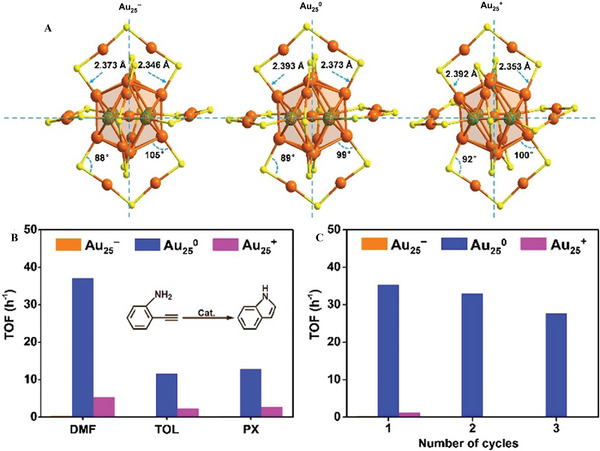
(A) Structure comparison of the Au_25_(SR)_18_ *^q^*. (B) Catalytic performances of the Au_25_(SR)_18_ *^q^* catalysts for intramolecular hydroamination of 2‐ethynylaniline. (C) Recyclability of the spent catalysts in *N*, *N*‐dimethylformamide (DMF) in turnover frequency. Adapted with permission.^[^ ^119^ ^]^ Copyright 2020, Wiley‐VCH. TOL, toluene; PX, paraxylene.

Li et al. researched reactivity and stability by regulating the free valence electrons of the Au 6s^1^ orbital inside and outside of Au_25_ *^q^* and explored its catalytic behavior for the intramolecular hydroamination of alkyne toward indole (Figure 11).^[^ ^119^ ^]^ The catalytic activity of Au_25_^0^ was the highest, followed by Au_25_^+^, and Au_25_^−^ was the lowest. Meanwhile, Au_25_^0^ showed higher stability compared to Au_25_^−^ and Au_25_^+^. The easy dissociation and subsequent agglomeration of AuSR in Au_25_^+^ are the main reason for the poor stability, while Au_25_^0^ and Au_25_^−^ with higher AuSR dissociation energy show relatively higher stability. The charge rearrangement between Au and the adsorbate promotes the dissociative adsorption of the reactants, accelerates hydroamination, and also enables intramolecular electron transfer from the adsorbate to facilitate intramolecular proton transfer. Intramolecular electron transfer makes Au_25_^+^ exhibit better activity than Au_25_^−^, but Au_25_^+^ has poor stability compared to Au_25_^−^. The charge difference of NCs has a significant impact on their catalytic performance and stability, which provides theoretical guidance for the structure‐activity relationship between free valence electrons and the physicochemical properties of NCs.

## 3 CONCLUSIONS AND PERSPECTIVES

This review highlights the most recent research progress in tailoring the catalytic activity and selectivity by precisely tuning the kernel structures of metal NCs, giving some insight into the influence of the size, and structural isomerism, of the metal NCs’ kernel structures on overall catalytic performance. Size‐dependent catalytic performance eventually began to study based on the successful synthesis of a series of Au*_n_* (SR)*_m_* NCs of different sizes. Although some interesting results have been made, more metal NCs of different sizes are still urgently needed for the in‐depth investigation of size‐dependent catalytic properties. The electronic properties of metal NCs can be modulated by the atom arrangement in the kernel, resulting in different catalytic performances. For example, Au_38_ and Au_36_ isomers as well as Au_28_ and Au_25_ quasi‐isomers, which have the same number of atoms but different atom arrangements, exhibit different catalytic activities. More interestingly, single‐atom dislodging/adding may have an important effect on the electronic structure of metal NCs, further showing an obvious change in catalytic properties. Therefore, the programmable kernel of metal NCs opens a new avenue for tailoring the catalytic performance on an atom‐by‐atom basis. Despite the advances in this field, there remain serious challenges including yield, stability, the blocked active metal sites by protected ligands, and the limited number of useful clusters, which further need more effort in the future to enable such catalysts to be used in industry. The purpose of this review is to look ahead to the potential challenges and opportunities in this emerging field, provide insightful guidance for the rational design and synthesis of atomically precise NCs catalysts, and lay a theoretical basis for understanding the correlation between structure and properties of NCs at the atomic level.

## CONFLICT OF INTEREST STATEMENT

The authors declare no conflicts of interest.
